# The ovarian transcriptome of the cattle tick, *Rhipicephalus (Boophilus) microplus*, feeding upon a bovine host infected with *Babesia bovis*

**DOI:** 10.1186/1756-3305-6-276

**Published:** 2013-09-23

**Authors:** Andrew M Heekin, Felix D Guerrero, Kylie G Bendele, Leo Saldivar, Glen A Scoles, Scot E Dowd, Cedric Gondro, Vishvanath Nene, Appolinaire Djikeng, Kelly A Brayton

**Affiliations:** 1 Knipling Bushland US Livestock Insect Research Laboratory, USDA-ARS, 2700 Fredericksburg Rd., Kerrville, TX 78028, USA; 2 Department of Mathematics, University of Texas at El Paso, El Paso, TX 79968, USA; 3 Animal Disease Research Unit, USDA-ARS, Pullman, WA 99164, USA; 4 Molecular Research, 503 Clovis Road, Shallowater, TX 79363, USA; 5 The Institute for Genetics and Bioinformatics, University of New England, Armidale, NSW 2351, Australia; 6 International Livestock Research Institute (ILRI) and Biosciences eastern and central Africa (BecA) Hub, PO Box 30709, Nairobi, Kenya; 7 Program in Vector-Borne Diseases, Department of Veterinary Microbiology and Pathology, Washington State University, Pullman, WA 99164, USA

**Keywords:** Cattle tick, *Rhipicephalus microplus*, *Babesia bovis*, Ovary, Transcriptome, EST

## Abstract

**Background:**

Cattle babesiosis is a tick-borne disease of cattle with the most severe form of the disease caused by the apicomplexan, *Babesia bovis*. Babesiosis is transmitted to cattle through the bite of infected cattle ticks of the genus *Rhipicephalus*. The most prevalent species is *Rhipicephalus (Boophilus) microplus*, which is distributed throughout the tropical and subtropical countries of the world. The transmission of *B. bovis* is transovarian and a previous study of the *R. microplus* ovarian proteome identified several *R. microplus* proteins that were differentially expressed in response to infection. Through various approaches, we studied the reaction of the *R. microplus* ovarian transcriptome in response to infection by *B. bovis*.

**Methods:**

A group of ticks were allowed to feed on a *B. bovis*-infected splenectomized calf while a second group fed on an uninfected splenectomized control calf. RNA was purified from dissected adult female ovaries of both infected and uninfected ticks and a subtracted *B. bovis*-infected cDNA library was synthesized, subtracting with the uninfected ovarian RNA. Four thousand ESTs were sequenced from the ovary subtracted library and annotated.

**Results:**

The subtracted library dataset assembled into 727 unique contigs and 2,161 singletons for a total of 2,888 unigenes, Microarray experiments designed to detect *B. bovis*-induced gene expression changes indicated at least 15 transcripts were expressed at a higher level in ovaries from ticks feeding upon the *B. bovis*-infected calf as compared with ovaries from ticks feeding on an uninfected calf. We did not detect any transcripts from these microarray experiments that were expressed at a lower level in the infected ovaries compared with the uninfected ovaries. Using the technique called serial analysis of gene expression, 41 ovarian transcripts from infected ticks were differentially expressed when compared with transcripts of controls.

**Conclusion:**

Collectively, our experimental approaches provide the first comprehensive profile of the *R. microplus* ovarian transcriptome responding to infection by *B. bovis.* This dataset should prove useful in molecular studies of host-pathogen interactions between this tick and its apicomplexan parasite.

## Background

The cattle tick, *Rhipicephalus (Boophilus) microplus*, is distributed worldwide and is detrimental to animal agriculture. Cattle producers incur substantial financial losses due to *R. microplus* infestations with Brazil alone experiencing losses of over $2 billion annually [1]. A substantial portion of these losses is attributable to pathogens and their associated diseases transmitted by the tick’s bite. The tick transmits two apicomplexan pathogenic agents, *Babesia bovis* and *Babesia bigemina*[2]. *B. bovis* is generally responsible for the more serious cases of bovine babesiosis, and frequently results in fatal infections of immunologically naive hosts. These pathogens infect the bovine erythrocyte, which is ingested by *R. microplus* during feeding upon an infected bovine host [2].

Following ingestion by the tick, merozoite stage apicomplexa undergo developmental changes until they are released from the bovine erythrocytes within the tick’s gut. After release, the apicomplexa complete their development to the zygote stage at which time they enter the digestive cells and begin multiplication and development until the kinete stage is reached. The apicomplexa then migrate from the digestive cells to the hemolymph and eventually spread to other tissues. Transmission of *Babesia* from adult tick to progeny is always trans-ovarian. After entering the developing oocytes, *Babesia* parasites undergo further development during the tick larval stage, and eventually occupy the tick’s salivary glands where they become infective to the vertebrate host [2].

Targeting ovarian proteins could adversely affect tick populations by causing a decrease in oogenesis and embryogenesis, thereby reducing reproduction rates, and by disrupting development and reproduction of disease causing *Babesia* parasites. Rachinsky *et al.*[3] showed that a number of *R. microplus* ovarian proteins are differentially expressed in response to *B. bovis* infection, including serine protease inhibitors, calreticulin, and peptidyl-prolyl cis-trans isomerases. These findings prompted the present study, as we wished to begin a characterization of the ovarian transcriptome, with emphasis on genes differentially expressed in response to ingestion of *B. bovis*-infected bovine blood. As our source of RNA, we used tissues that were used in the ovarian proteome study of Rachinsky *et al.*[3] to allow a direct comparison between the proteomic and transcriptomic response.

In the Rachinsky study, *R. microplus* fed upon a splenectomized calf suffering from bovine babesiosis due to infection with *B. bovis*. For the current study, we obtained dissected *R. microplus* ovaries archived from their study and compared gene expression in ovary dissected from adult female ticks which had fed on the infected calf with gene expression in corresponding tissue from ticks at a similar developmental stage that fed on an uninfected control calf. Our approaches included sequencing a subtracted library synthesized from infected ovarian mRNA, microarrays, serial analysis of gene expression (SAGE), and quantitative real-time polymerase chain reaction (qRT-PCR) to identify ovarian transcripts differentially expressed in association with *B. bovis* infection of *R. microplus*.

## Methods

### Animal use protocol

All animal use was conducted at ADRU facilities at the University of Idaho Holm Research Center (Moscow, ID) while following protocols approved by the University of Idaho Institutional Animal Care and Use Committee.

### Tick strain

The ticks were taken from the f20 generation of the La Minita strain of *R. microplus*, which has been maintained as a *Babesia*-free laboratory colony at The University of Idaho Holm Research Center since 1999. La Minita was originally collected from an outbreak in Starr County, Texas in 1996 and propagated at the USDA Cattle Fever Tick Research Laboratory at Moore Field, Texas. All calves used in this study were splenectomized Holstein breed and 5–6 months of age.

### Sample collection

Tissues used in the transcriptome studies were the same as those obtained and dissected for the ovarian proteome study of Rachinsky *et al.*[3]. During the tissue dissection stage of that study, samples had been randomly assigned for either a proteome study or a transcriptomic study. The procedures for obtaining *B. bovis*-infected and uninfected adult stage engorged females were reported by Rachinsky *et al.*[3]. Briefly, for the uninfected ticks, larvae from 1 g of *R. microplus* eggs were placed on a calf on study day 1 and replete female ticks began dropping on study day 22 and continued dropping until study day 31 when the animal was euthanized. The ovaries for the uninfected sample were obtained from female ticks that dropped on day 22 and were maintained for 4 days at 23°C at which time oviposition began and the ticks dissected within 24 hr. During collections of the *B. bovis*-infected ticks (described below), it was noted the period from when the replete females dropped from their bovine host to when ovipositioning began was twice as long compared to the uninfected ticks. Thus, we utilized the onset of oviposition as our reference point to biologically synchronize the dissection timings between the two samples.

To obtain *B. bovis*-infected ticks, two splenectomized calves were infested with tick larvae as above and the calves were infected on day 14 with *B. bovis*. This was done by intravenous inoculation with 1.8 ml of blood stabilate culture originating from the T2Bo strain of *B. bovis*, stored in liquid nitrogen and routinely verified as highly infective to bovines. The calf infections were monitored by daily measurement of rectal temperature, which peaked on day 22, and both animals were required to be euthanized on study day 24 due to the progression of babesiosis. All collected female ticks were incubated at 23°C for nine days, at which time oviposition and dissections commenced.

Riek [4] reported that 4–5 days after females have fed to repletion on a *B. bovis*-infected splenectomized calf, *B. bovis* vermicules can be observed in mature ova. Thus, a 9 day incubation period of the females collected from the *B. bovis*-infected calves should have produced infected ovaries. We confirmed ticks were infected with *B. bovis* by examining hemolymph smears from 66 randomly selected ticks that dropped on study day 24 from the two infected calves. Forty-six ticks had at least 5 *B. bovis* kinetes per high power microscopic field and the other 20 ticks had 3–5 kinetes per high power field.

SAGE was used as a third transcriptomic protocol to complement the subtracted library and microarray analyses of infection-induced differential gene expression. In the SAGE experiment on *B. bovis*-infected ovaries, dissections were performed using engorged female ticks that had dropped after feeding to repletion on the *B. bovis*-infected calves as described above. After dropping, the engorged ticks were incubated at 23°C for either 0 or 6 days prior to dissection.

Because of the status of *R. microplus* as an arthropod requiring adherence to strict USDA quarantine and handling restrictions for *B. bovis*-infected experimental calves requiring the need to sacrifice the calves at the end of each experiment, the ideal of using independent biological replicates was not met. Animal experiments were approved by the Institutional Animal Care and Use Committee at Washington State University, USA, in accordance with institutional guidelines based on the U.S. National Institutes of Health (NIH) Guide for the Care and Use of Laboratory Animals.

### RNA protocols

The Totally RNA Kit (Ambion Inc., Austin, TX, USA) was used to purify RNA from tick ovaries obtained from 20 ticks with a final lithium chloride precipitation step added per kit protocol booklet. The ovary RNA was obtained by dissecting individual engorged females into RNA*later* (Ambion Inc.) and the dissected materials were pooled prior to isolating total RNA. Total RNA was treated with Turbo DNAse as per Turbo DNA-free kit protocols (Ambion Inc.). RNA integrity was verified by formaldehyde gel electrophoresis and staining in GelStar Nucleic Acid Gel Stain (Lonza, Rockland, ME, USA).

### Subtracted and SAGE library synthesis

Two 250 μg samples, of *B. bovis*-infected or uninfected ovary total RNA, were sent to Express Genomics Inc. (Frederick, MD, USA) for subtracted library synthesis. Primary libraries were amplified from the uninfected and *B. bovis*-infected material, following directional cloning into the pExpress-1 vector digested with NotI and EcoRV. Subsequently, a subtracted library was produced by subtracting the *B. bovis*-infected material with the uninfected material, enriching for expressed genes in the *B. bovis*-infected material. Express Genomics quality control checks found a 100-fold reduction in the number of clones that hybridized to actin in the subtracted library compared with the primary library, verifying the subtraction process was successful. The SAGE libraries were synthesized from 50 μg of total RNA from engorged female tick ovaries using the I-SAGE Long Kit as per manufacturer’s protocols (Invitrogen Inc., Carlsbad, CA).

### Transcriptome sequencing

EST and SAGE library sequencing was performed at the J. Craig Venter Institute (Rockville, MD). Bacterial colonies were picked for template preparation using colony-picking robots (Genetix, Boston, MA), inoculated into 384 well plates containing liquid medium and incubated overnight at 37°C. A robotic workstation was used to prepare sequencing grade plasmid DNA via an alkaline lysis method modified for high throughput processing [5]. Beckman Multimek 96 or Biomek FX automated pipetting robot work stations (Beckman Coulter, Fullerton, CA) were used to combine pre-aliquoted templates and sequencing reaction mixes. Linear amplification steps were performed on MJ Research Tetrads PTC-225 (MJ Research, Inc., Watertown, MA) and sequencing reaction products purified by ethanol precipitation and analyzed on ABI 3730xl sequencing machines (Applied Biosystems, Foster City, CA). The unassembled ovary subtracted library EST sequences were submitted to GenBank dbEST (GenBank: FG301341-FG305398). SAGE library sequences were extracted and analyzed by a set of custom-written perl scripts.

### Bioinformatics analysis

Sequence assembly and annotation were performed as described in Heekin *et al.*[6]. Briefly, several screening steps were applied to eliminate contaminated or low quality sequences from the subtracted library prior to assembly. A *de novo* transcript assembly was performed on the subtracted library using cap3 [7]. All resulting contigs and unassembled singletons (collectively referred to as unigenes) were used in subsequent analyses (Additional file 1). Annotations were initially assigned to unigenes using similarity search methods of the Uniref100 database using BLASTX with an *e*-value cutoff of 1e-07 [8]. Sequences with no BLASTX high-scoring pairs (HSPs) were submitted to the prot4EST application to predict the correct open reading frame (ORF) for each sequence [9]. After the ORFs were predicted, the sequences were submitted to annot8r for assignment of Gene Ontology (GO) terms [10,11].

### Microarray design

Twenty μg of each DNA-free total RNA was sent to NimbleGen Systems Inc. (Madison, WI, USA) for use in microarray hybridization. A custom high-density single channel oligonucleotide array was designed by NimbleGen Systems Inc. using 13,601 of the 13,642 members of BmiGI Version 2 and these arrays were described in detail by Saldivar *et al.*[12].

Our array experimental design consisted of four replicates, two replicate microarrays for each of the infected and uninfected samples. Also, in the array design, each transcript represented on the array has 14 different 50-mer probes, establishing an additional layer of replication. Each probe is spotted twice on each array as technical replicates. Sample labeling, hybridization, array scanning, and image analysis was performed at NimbleGen Systems Inc. as described by Saldivar *et al.*[12]. Quality control measures and pre-processing were performed using the computing language R [13] and Bioconductor [14]. The quality of the arrays was assessed through standard quality control measures: pseudo-images of the arrays to detect spatial effects, scatter plots of the arrays versus a pseudo-median reference chip and summary statistics including histograms and boxplots of raw and normalized log intensities. All microarray quality control measurements were within recommended limits as established and implemented by Nimblegen.

Gene calls were generated and normalized as described [6]. The microarray data have been submitted to the GEO database (http://www.ncbi.nlm.nih.gov/geo/; GEO accession number GSE10816). Significance Analysis of Microarrays (SAM) [15,16] was performed in the Microarray Experiment Viewer (MeV Version 4.0, Dana-Farber Cancer Institute, Boston, MA, USA) to select statistically significant differentially expressed genes. The design used by SAM is a two-class unpaired design, where samples fall in either the infected or uninfected group. The cutoff for significance is determined by a tuning parameter delta and a minimum fold change threshold to ensure that called genes change at least a specified amount. The threshold value delta was set to 0.53 and fold change set to ≥ 2.0. Because of the unavailability of biological replicates, the *p*-values and *d* statistics related to the microarray data should not be interpreted as statistical probabilities. Nevertheless, these statistics remain useful for prioritizing candidates for comparison with the SAGE and subtracted library results.

### Verification by real-time PCR

Array results were verified for three target genes based on their level of differential expression and the amount of annotation available for their corresponding BmiGI sequence. The same total RNA samples used for the microarrays were also used for quantitative real-time PCRs. The RETROscript Kit Reverse Transcription for RT-PCR (Ambion) was used as per manufacturer’s recommendations to produce cDNA from four micrograms of DNA-free total RNA for each sample. Primers and TaqMan probes were designed using Beacon Designer 7.5 (PREMIER BioSoft International, Palo Alto, CA; Additional file 2) and synthesized by Sigma-Aldrich Inc. (Atlanta, GA) for each gene selected and for the *R. microplus* 18S rRNA gene, which was the reference gene for normalization [12]. Validation experiments were run on each gene and the reference gene to determine PCR efficiencies and optimal concentrations.

All real-time reactions were carried out in clear low-profile 96 well plates (no. MLL9601, BioRad, Hercules, CA). The 25 μL total reaction volumes included primers, 250nM TaqMan probe, TaqMan Universal Master Mix No AmpErase UNG (Applied Biosystems Inc., Foster City, CA) and corresponding RETROscript cDNA. The final primer concentration for the 18S rRNA reference gene and the targeted genes was 900nM for both the forward and reverse primers. The BioRad CFX96 Real-Time System was used with a cycling protocol of 95°C for 10 min, and 50 cycles of 95°C for 15 sec, 60°C for 1 min plus plate read. The fluorescence emission data analysis was done using baseline subtracted curve fit mode with CFX Manager Software version 1.0 (BioRad). All primer and probe sequences are listed in Additional file 2.

## Results and discussion

### Subtracted library results

Over 4,100 bacterial colonies from the subtracted library were prepared for sequencing and this resulted in 4,045 high quality tick EST sequences. This dataset assembled into 727 unique contigs (clusters of related transcripts) and 2,161 singletons (transcripts that did not cluster) in two separate passes for a total of 2,888 unigenes (Additional file 1). Contigs that clustered during the first pass received the prefix contigA. Contigs from the first pass that clustered with additional sequences during the second pass of the assembler received the prefix contigB. Singleton sequences retained their original labels assigned during sequencing. The mean unigene length was 851.7 nucleotides. Out of 2,888 unigenes, approximately one-third received significant BLASTX HSPs (*e*-value < 1e-07) from the Uniref100 database and are listed in Additional file 3. The ORFs for all unigenes predicted by prot4EST are listed in Additional file 4. BLAST results reported in this study are from BLASTX searches of the Uniref100 database unless otherwise noted.

**Figure 1:**
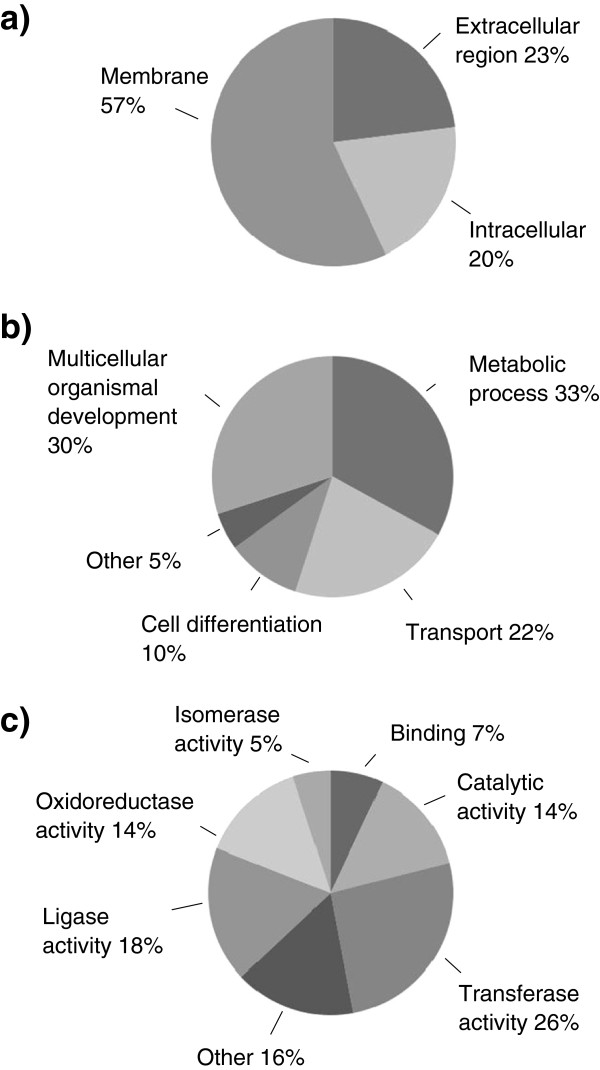
**High level GO classification of unigenes from the subtracted library. a**: Cellular Component Ontology, **b**: Biological Process Ontology, **c**: Molecular Function Ontology.

Figure 1 lists a summary of the GO annotation by the annot8r application. The unigenes are categorized by three ontology domains consisting of 29 high-level GO terms. In the cellular component domain (C), most of the differentially expressed transcripts (57%) were classified as membrane. A number of unigenes, however, were classified as extracellular (23%) or intracellular (20%). The majority of annotations in the molecular function domain (F) were assigned the GO terms: transferase activity (26%), ligase activity (18%), oxidoreductase activity (14), and catalytic activity (14%). In the biological process domain (P), metabolic process (33%), multicellular organismal development (30%), and transport (22%) were predominant. The complete GO annotation set is listed in Additional file 5.

#### ***Genes related to stress, detoxification, and immune response***

The subtracted library BLASTX analysis identified a number of unigenes with the GO term “stress response” (Table 1; Additional file 3). There were 12 unigenes with sequence similarity to cytochrome P450s, which are a group of enzymes that catalyze metabolism of organic molecules including toxins and xenobiotics. Transcripts encoding superoxide dismutase were found in the subtracted library (ContigA418 and MPOA822TR). Additional detoxification proteins related to glutathione metabolism were also observed, including ContigA295, which is a glutathione *S*-transferase (GST). GST expression has been previously induced upon blood feeding in *R. microplus*, and is postulated to be an adaptive response to reactive oxygen species created during the blood meal [17]. GSTs may also facilitate digestion of the meal by reducing proteins and lipids [18]. Additional unigenes were similar to other protein-reducing antioxidants including glutaredoxin (ContigA561), peroxiredoxin (MPOAE24TR), and several peroxinectins (ContigA694, MPOA737TR, MPOA895TR, MPOA475TR, MPOAC37TR and MPOAK85TR). The library also contained 5 stress-induced heat shock protein transcripts, ContigA105, ContigA652, MPOAC82TR, MPOAF39TR. The subtraction library contained two cytochrome *c* oxidase ESTs (MPOAA34TF and MPOAN31TR), and these proteins have been indirectly linked to hemocyte modifications occurring during *Borrelia burgdorferi* infection of *Ixodes ricinus*[19].

**Table 1:** Unigenes from subtracted library annotated by BLASTX and with GO term of stress response

**Unigene **^**a**^	**Protein annotation**	**Species**	**Acc. no.**	***e*-value**^**b**^
ContigA171	Cytochrome P450	*Tribolium castaneum*	D6W6R3	1e-68
ContigA591	Cytochrome P450	*Ixodes scapularis*	B7PT10	6e-12
MPOAJ93TR	Cytochrome P450	*Ixodes scapularis*	B7PN37	2e-45
MPOAN40TR	Cytochrome P450	*Ixodes scapularis*	B7PJW2	5e-24
MPOAH45TF	Cytochrome P450	*Ixodes scapularis*	B7QJP3	5e-80
MPOAG88TR	Cytochrome P450	*Ixodes scapularis*	B7P5V0	1e-47
MPOAN40TF	Cytochrome P450	*Ixodes scapularis*	B7PTT2	4e-43
MPOAG88TF	Cytochrome P450	*Ixodes scapularis*	B7PME9	6e-32
MPOAG70TR, MPOAH45TR	Cytochrome P450	*Ixodes scapularis*	B7QJP2	6e-78
ContigA57, ContigA143	Cytochrome P450	*Ixodes scapularis*	B7P5I8	1e-124
ContigA418	Superoxide dismutase	*Amblyomma maculatum*	G3MQI9	9e-89
MPOA822TR	Superoxide dismutase	*Amblyomma maculatum*	G3MQ28	2e-108
ContigA652	Heat shock protein	*Aedes aegypti*	Q17PR3	7e-135
MPOAC82TR, MPOAC82TF	Heat shock protein 70	*Moina mongolica*	D2E4A4	4e-103
ContigA105	Heat shock protein	*Ixodes scapularis*	B7QJZ5	3e-32
MPOAF39TR	Heat shock protein	*Ixodes scapularis*	B7PAR6	3e-144
ContigA295	Glutathione S-transferase	*Ixodes scapularis*	B7Q9K1	3e-143
ContigA561	Glutaredoxin	*Ictalurus furcatus*	E3TDC4	1e-38
MPOAE24TR	Peroxiredoxin	*Ixodes scapularis*	B7Q7K9	6e-44
MPOAA34TF	Cytochrome *c* oxidase assemby	*Aedes aegypti*	Q16M46	4e-40
MPOAN31TR	Cytochrome *c* oxidase assembly	*Ixodes scapularis*	B7P8T9	9e-111
ContigA694, MPOA737TR, MPOA895TR	Peroxinectin	*Ixodes scapularis*	B7P9B9	2e-12
MPOA475TR	Peroxinectin	*Ixodes scapularis*	B7PUM7	2e-116
MPOAK85TR, MPOAC37TR	Peroxinectin	*Ixodes scapularis*	B7PQ34	1e-84

^a^ Unigene identification number as listed in Additional file 1.

^b^ In the case of multiple *e*-values obtained from multiple unigenes, the highest *e*-value is listed.

Table 2 lists unigenes annotated with several additional stress- or immune-related GO terms including four unigenes with the GO term “response to DNA damage stimulus” (ContigA564, MPOAC91TR, MPOAF62TR, MPOAG90TR). Unigenes MPOAF05TR and ContigA290 were annotated with the GO term “carboxylesterase activity”. Carboxylesterases were induced in the midgut of the silkworm in response to insecticides [20] and are involved in the acaricide resistance mechanisms found in *R. microplus*[21]. Unigenes ContigA711, MPOAC37TR, and MPOAK85TR received the GO term “response to oxidative stress”. The unigene MPOA256TR was annotated with the GO term “defense response to bacterium” but did not have a useful hit in the UniRef100 database. Unigenes MPOAL11TR and ContigA709 received the GO term “immune response” but were annotated only as uncharacterized proteins.

**Table 2:** Unigenes from subtracted library annotated with stress and immune-related GO ontology terms

**Unigene **^**a**^	**GO accession no.**	**Category**^**b**^	**GO annotation**
ContigA290	GO:0004091	F	Carboxylesterase activity
MPOAF05TR	GO:0004091	F	Carboxylesterase activity
ContigA711	GO:0006979	P	Response to oxidative stress
MPOAL11TR	GO:0006955	P	Immune response
ContigA709	GO:0006955	P	Immune response
ContigA564	GO:0006974	P	Response to DNA damage stimulus
MPOAC91TR	GO:0006974	P	Response to DNA damage stimulus
MPOAF62TR	GO:0006974	P	Response to DNA damage stimulus
MPOAG90TR	GO:0006974	P	Response to DNA damage stimulus
MPOA256TR	GO:0042742	P	Defense response to bacterium

^a^ Unigene identification number as listed in Additional file 1.

^b^ GO category F = function, P = process.

Proteases and protease inhibitors were markedly induced in the ovary and possibly contribute to the ovarian immune response. Many of these catabolic enzymes included defensin-like molecules, including serpins, cathepsins, legumain, and microplusin (Additional files 1 and 3). Defensins are small catabolic peptides with specific antimicrobial activity. Unigene ContigA321 was orthologous to a putative defensin in *I. scapularis*. Defensin was induced in the gut of *D. variabilis* when challenged with *Borrelia burgdorferi*, but was not induced in the same tick when challenged with two different species of Gram-positive bacteria [22]. ContigA321 did not show significant sequence similarity to any of the ovary up-regulated transcripts reported by Stutzer *et al.*[23] nor to ESTs reported from other tick species. A 687 bp EST from an embryonic cell line of *R. microplus*, GenBank Accession EW679737, was identical to the 1188 bp ContigA321 (data not shown). Thus ContigA321 may represent a defensin unique to ovarian or embryonic tissue. Unigenes MPOA768TF and TR showed high similarity to a putative legumain-like protease from two species of ticks. Legumain is an asparaginyl endopeptidase that processes microbial antigens in lysosomes [24]. Microplusin is a unique cysteine-rich secreted antimicrobial peptide (AMP), which is active against bacteria and fungi [25]. In addition to being found in the subtracted library ContigB21, the transcript encoding microplusin was also one of the most abundant seen in the SAGE experiment (described below). Microplusin transcript levels in the ovaries of *R. microplus* gradually rise before peaking at the beginning of oviposition [26]. Microplusin was also among the up-regulated transcripts in a transcriptome experiment that examined gene expression in *B. bovis*-infected larvae of *R. microplus*[6].

The subtraction library contained several transcripts that may participate in the tick immune response (Additional files 1 and 3). The unigene MPOAB56TF was annotated as tumor necrosis factor receptor-associated factor (TRAF), which is an important regulator of inflammation, apoptosis, and antiviral responses (Additional file 3). ContigA62 was an ortholog to a putative alpha-2-macroglobulin in *I. scapularis*, which was up-regulated in *Dermacentor variabilis* when exposed to *Anaplasma marginale*[27]. Unigene MPOAA26TF showed significant similarity to fucosyltransferase, which has been demonstrated to increase microbial pathogenesis in *I. scapularis*[28]. Putative ixoderins were also identified (MPOAH63TR, ContigA104, and ContigA667); ixoderin is a lectin-like molecule with a possible role in innate immunity in ticks [29].

Serpins were the predominant protease inhibitor in the subtracted library. Serpins regulate blood coagulation cascades, transport of hormones and are components of the immune system of many invertebrates [30]. Serpins have significant roles in antimicrobial and antifungal responses in insects [31]. ContigA484 had sequence similarity to a cysteine peptidase inhibitor in *M. musculus*. A recent review of tick cysteine protease inhibitors (cystatins) characterized their roles in detoxification, innate immunity regulation, pathogen transmission and immunosuppression [32].

Cathepsins, which comprise cysteine, aspartic, and serine proteases, were the dominant protease family in the subtracted library. Cysteine proteases are important constituents of the immune response of *R. microplus* and participate in vitellin degradation [33,34]. The unigene MPOAH54TR had high similarity to a cysteine protease, longipain. This enzyme was recently characterized in the babesial parasite vector tick *Haemaphysalis longicornis*. Longipain was specifically localized to lysosomal vacuoles and was shown to be a potent parasiticide [35]. Aspartic proteases have been linked to digestion and vitellin degradation in ticks [36]. Three of the induced proteases, ContigA110, MPOA037TF, and MPOAM53TR, were serine-type proteases. Three serine protease transcripts were up-regulated in ovary tissues of *R. microplus*[23]. However, there was no significant sequence similarity between these ovarian serine proteases and those reported here (data not shown). Serine-type proteases may be involved in vitellin degradation, which was inferred from a study of a trypsin-like serine peptidase expressed in lice embryos [37]. Serine proteases from the gut of *H. longicornis* were also up-regulated during the blood-feeding process [38].

### Microarray results

Our microarray approach compared the ovary transcriptome from engorged ovipositing females that fed on a *B. bovis*-infected bovine host to the ovary transcriptome from ticks fed on an uninfected host. The microarray experiments identified 15 transcripts that were expressed at a higher level in ticks feeding upon a *B. bovis*-infected calf compared with ticks feeding on an uninfected calf (Table 3; Additional file 6). No transcripts were found to be statistically expressed at a lower level in ovaries from adult female ticks feeding upon a *B. bovis*-infected calf compared with ovaries from ticks feeding on an uninfected calf. As similarly reported by Saldivar *et al.*[12] and Stutzer *et al.*[23], a number of the differentially expressed tick genes had no useful annotation; six of the fifteen transcripts in Table 3 did not have significant (e < 0.001) BlastX hits.

**Table 3:** ***R. microplus *** **genes with highest up-regulation associated with *B. bovis *** **infection in microarrays**

**ID**^**a**^	**d**^**b**^	**FC**^**c**^	**BlastX annotation**			
			**Protein name**	**Species**	**Acc. no.**	***e*-value**
**TC12551**	2.1	47.8	put. secreted salivary gland peptide	*Ixodes scapularis*	XP002411978.1	3e-04
**TC9311**	1.9	60.8	Kunitz-like protease inhibitor	*Ancylostoma caninum*	AAN10061.1	5e-105
TC6492	1.9	32.8	Kunitz-like protease inhibitor 6	*Rhipicephalus microplus*	P83606.2	0.0
**TC13077**	1.9	39.6	Kunitz-like protease inhibitor	*Ancylostoma caninum*	AAN10061.1	5e-116
TC9020	1.8	30.8	NSS^d^	*-*	-	-
**TC6491**	1.5	16.1	Kunitz-like protease inhibitor 6	*Rhipicephalus microplus*	P83606.2	2e-94
**TC6326**	1.5	16.6	put. secreted salivary gland peptide	*Ixodes scapularis*	XP002411978.1	3e-10
BEAAA85TR	1.5	19.9	NSS	*-*	-	-
TC11578	1.4	16.9	NSS	*-*	-	-
**BEABQ71TR**	1.2	10.5	GGY domain protein	*Amblyomma variegatum*	DAA34729.1	1e-09
BEAC749TR	1.2	10.1	NSS	*-*	-	-
**TC6758**	1.1	7.9	hypo. protein IscW	*Ixodes scapularis*	XP002411179.1	1e-54
TC8946	1.1	7.9	NSS	*-*	-	-
TC6671	1.1	8.9	NSS	*-*	-	-
TC5979	1.0	7.5	hypo. conserved protein 57	*Amblyomma variegatum*	DAA34262.1	2e-33

Gene IDs in bold type are also represented in the subtracted library.

^a^ The identification number from BmiGI Version 2 (http://compbio.dfci.harvard.edu/tgi/cgi-bin/tgi/gimain.pl?gudb=b_microplus).

^b^ d is the d statistic as performed by SAM.

^c^ FC is the fold change ratio.

^d^ No statistically significant similarity found in BlastX search, based on *e*-value < 0.001.

Four of the most up-regulated transcripts in the adult female ovary microarrays had high sequence similarity to Kunitz-like protease inhibitors (Table 3). Kunitz-type inhibitors have been studied in *R. microplus* and exhibit activity against bovine trypsin and human neutrophil elastase [39]. These inhibitors have also been shown to be differentially expressed in tick salivary glands in response to pathogen infection [40]. Thus, Kunitz-type inhibitors likely play a defensive role in the tick ovary. Four Kunitz-type protease inhibitors were found in the subtracted library dataset (Additional file 3), thus corroborating the microarray findings.

To verify the microarray results, we selected 3 transcripts with differential expression of varying fold-changes and performed qRT-PCR to compare transcript levels in the *B. bovis*-infected ovary tissue with the uninfected control (Table 4). The directional expression changes for all selected transcripts were qualitatively similar in both the microarrays and qRT-PCRs. In fact, the three transcripts showed higher differential regulation in the RT-PCR than in the microarrays, which was expected since array results were compressed towards zero during their analysis.

**Table 4:** RT-PCR Verification of selected microarray results

	**Microarray**^**a**^		**Relative quantitative PCR**^**a**^	
**EST**	**Uninfected**	**Infected**	**Uninfected**	**Infected**
TC9020	1	30.8	1	395
TC9311	1	60.8	1	206
TC13077	1	39.6	1	61

^a^ Normalized data to set lower value to 1 for comparison purposes.

### SAGE results

SAGE was used to identify genes that are responding to the infection process whereby *B. bovis* enters and replicates in the tick ovary. SAGE libraries were produced from ovaries dissected from engorged female ticks the day of dropping from the bovine host (designated day 0) and 6 days after dropping. Since the subtracted library and microarray protocols assessed the ovarian transcriptome 9 days post-repletion, the time period between repletion and the onset of oviposition was investigated using SAGE. The 6 day time point was selected because Riek [4] reported that *B. bovis* vermicules could be found in mature ova 4–5 days after females fed to repletion. Additional files 7 and 8 contain the complete raw data sets for SAGE tag counts and associations between SAGE tags. Table 5 shows the numbers of tags in both libraries, the number of unique tags and their distribution into abundance classes. Combining both libraries, 792 of the tags had exact matches to BmiGI Version 2.0 in the forward direction (Additional file 9) and 568 of the tags had matches in the reverse complement direction (Additional file 10). Tables 6 and 7 present the 50 most abundant SAGE tags in the control and infected libraries, respectively. Each tag’s corresponding match to BmiGI Version 2 is indicated and annotation is given if available. Twenty-two tags from the Control (Day 0) library and 16 tags from the Infected (Day 6) library had useful annotation. Table 8 lists the tags with >10-fold differential expression comparing the Control and Infected SAGE libraries. Twenty-four tags were in the up-regulated category while 13 tags were in the down-regulated category. Seven of the up-regulated tags and 6 of the down-regulated tags in this table had an exact match to a member of BmiGI Version 2.0. All up-regulated and down-regulated tags are listed in Additional files 11 and 12, respectively.

**Table 5:** Number of LongSAGE tags and abundance classes from each library

**% Abundance**	**Overall number of tags**		**Number of unique tags**	
	**Control**	**Infected**	**Control**	**Infected**
> 1.0	0	481	0	2
0.2 - 1.0	1032	903	39	32
0.05 - < 0.2	1429	1110	227	143
< 0.02	1152	1907	516	789
0.02 - < 0.05	3151	4349	3151	4349
Total	6764	8750	3933	5315

**Table 6:** The 50 most abundant SAGE tags in the pooled tag data from control LongSAGE library

**Rank**	**Tag**^**a**^	**Count**	**Infected**	**BmiGI match**			
			**Library rank**	**Description**	**Species**	**e-value**	**ID**
1	ACGTGACTGTCGCCACC	57	29	-	-	-	-
2	TGGTGCCCGAAACGAAG	55	106	Uro-adherence factor A	*Taloromyces stipitatus*	1e-05	TC12322
3	TGGCTGGCTGCCCACTG	48	18	Ribosomal protein P0	*Haemaphysalis longicornis*	2e-35	TC9039
4	GACGGCGAGTGGGAACC	45	116	calreticulin	*Rhipicephalus microplus*	1e-165	TC8950
5	TACGAAGCGCTGGCAGA	43	49	Disulfide isomerase	*Haemaphysalis longicornis*	4e-48	TC8716
6	ACGCGACTGTCGCCACC	40	29	Putative secreted protein	*Ixodes scapularis*	3e-72	TC5798
7	TCTGGACGCGGCAAGGG	38	425	-	-		-
8	GCCCGCAGCGGCTGAAC	35	50	Hypothetical protein	*Ixodes scapularis*	2e-10	TC7866
9	CTCACCGACCCGTCGGC	35	43	-	-	-	-
10	GGTCCACCCCAGCGACT	33	31	Hypothetical protein	*Ixodes scapularis*	8e-104	TC12372
11	CGCAAGGCCCAAGGAGG	29	260	Hypothetical protein	*Ixodes scapularis*	1e-20	TC9377
12	CGCAAGGCCCAAGGAGG	29	50	-	-	-	-
13	GTGGTGCACGCCAACCC	28	80	Superoxide dismutase-	*Apis mellifera*	8e-44	TC12062
14	CCAGCGCTAAAGATGCG	27	59	-	-	-	-
15	GAGGCGGTGCGGGAGAG	27	178	-	-	-	-
16	GCACGGCGATGCGACGG	26	39	-	-	-	-
17	TGTGGCTGGGGCTCCGC	26	50	-	-	-	-
18	GACTCCAATGAAGGCCC	25	25	Alpha tubulin	*Mus musculus*	0.0	TC9399
19	ACACGACTGTCGCCACC	23	116	-	-	-	-
20	GCGAGGAGCTTGTCGGG	22	35	-	-	-	-
21	GCTGTGGTTGCGCGCAC	22	59	-	-	-	-
22	CTGCAGACGTTGACGGG	21	97	Adipose differentiation related protein	*Ixodes scapularis*	8e-75	TC9098
23	GGCCCCCTCCCGCCCAA	21	No^b^	Transaldolase	*Macaca mulatta*	5e-57	TC13335
24	TGCGCAAAGGACGCCCG	21	178	Serine proteinase inhibitor	*Rhipicephalus microplus*	1e-26	BEAET94TR
25	ATCTGAGTTTAGACCGA	21	1	Mitochondrial DNA	*Rhipicephalus sanguineus*	0.00	TC5761
26	CTGAGGATTGCCGAGCC	20	97	60S ribosomal protein L7	*Argas monolakensis*	1e-104	TC5935
27	CACGTACAACCTCTGCG	19	No	-	-	-	-
28	GGGGAGTCTGACGACTG	19	80	-	-	-	-
29	GGCGACCGCTTCACCGA	19	68	Myosin regulatory light chain		5e-83	TC12444
30	TTGTGCAGCGATCGGCA	19	425	-	-	-	-
31	GCACCTGGCGCTGGCAA	18	260	-	-	-	-
32	CTGGCCGCTTGGGTCCG	18	116	-	-	-	-
33	GTAGGCCCGGTATTGGT	17	18	-	-	-	-
34	CACCTTTGCATCGACGC	15	68	-	-	-	-
35	GTACCAGAGGACAAGCC	15	967	-	-	-	-
36	GGAAGCGCTAAGCGGCC	14	50	-	-	-	-
37	GAGGCACAGGCGCCGAA	14	143	60S ribosomal protein L13e	*Amblyomma americanum*	3e-85	TC12299
38	TCTGTGCGTGCCAAGGA	14	116	60S ribosomal protein L10	*Ixodes scapularis*	2e-48	TC8894
39	GTCAGCTGATGGGCAGA	14	178	G nucleotide binding protein	*Dermacentor variabilis*	2e-167	TC6908
40	CAAATCTCTGCGTGGCA	13	260	Translation initiation factor 2C	*Ixodes scapularis*	4e-27	TC6114
41	GCCTGCGTTTGCTGCAG	13	143	Nucleolysin RNA binding protein	*Pediculus humanus corporis*	2e-122	TC12242
42	TTGCGGCTGCGCCGCAC	13	260	Golgi protein involved in ER retention	*Ixodes scapularis*	2e-81	TC12211
43	GTTTGTGAGAGCGCCGC	13	260	-	-	-	-
44	CCCGCGGTCATCACGGA	13	143	-	-	-	-
45	AAGGCGCCAGCGGTGAT	13	68	-	-	-	-
46	GCCGCACACTTTGACAG	12	97	Ubiquitin/ribosomal protein S27A	*Dermacentor variabilis*	1e-61	TC10071
47	GGTTGGGCGCCGACGCG	12	178	Ubiquitin protein ligase	*Ixodes scapularis*	1e-76	BEABI57TR
48	GCGTTTGCTGGTGCCAG	12	178	Maleate dehydrogenase	*Ixodes scapularis*	2e-164	TC9744
49	TGGTGGTAGCTGGTGCG	12	14	-	-	-	-
50	GTGGTGCCGTCGGCGCT	12	260	-	-	-	-

^a^ CATG trimmed from each tag’s 5′ end for clarity.

^b^ Indicates tag not found in Infected library.

**Table 7:** The 50 most abundant SAGE tags in the pooled tag data from Infected LongSAGE library

**Rank**	**Tag**^**a**^	**Count**	**Control**	**BmiGI match**			
			**Library rank**	**Description**	**Species**	***e*-value**	**ID**
1	ATCTGAGTTTAGACCGA	339	25	Mitochondrial DNA	*Rhipicephalus sanguineus*	0.00	TC5761
2	TGATTGTGTGCTATGTG	142	783	-	*-*	-	
3	GGGGCAAACACTATGGA	85	No^b^	-	*-*	-	
4	AAGATCACACTGGCATT	57	783	-	*-*	-	
5	TTTTCCCCAACCCAGGA	50	No	Microplusin	*R. microplus*	7e-49	BEACP61TR
6	CAGGCTGTCCCAGCAAT	43	267	Secreted salivary gland peptide	*I. scapularis*	1e-05	BEAE009TR
7	TTTTTCCCAACCCAGGA	39	783	-	*-*	-	
8	GGTCAAGGGGTAATAAA	37	No	-	*-*	-	
9	GTGGTTACGGAGGCGGG	36	No	-	*-*	-	
10	GACGGCCCTTGCAAGTG	33	128	-	*-*	-	
11	CAGAAGCTTCAAAGCCA	33	783	-	*-*	-	
12	TCGACAGGGTCATTCCG	31	No	-	*-*	-	
13	GCCGTTCTTAGTTGGTG	28	783	-	*-*	-	
14	TGGTGGTAGCTGGTGCG	25	49	-	*-*	-	
15	GCGGTTACGGAAGCGGG	25	No	-	*-*	-	
16	CAGTTGTTGTTGCAGGG	24	77	-	*-*	-	
17	AAGATCACGCTGGCATT	24	No	-	*-*	-	
18	GTAGCCGCCAGCCAAGG	22	387	-	*-*	-	
19	TGGCTGGCTGCCCACTG	22	3	Ribosomal protein P0	*Haemaphysalis longicornis*	2e-36	TC9039
20	GTAGGCCCGGTATTGGT	22	33	-	*-*	-	
21	CACATCATAGAACAGCT	21	No	-	*-*	-	
22	CTGTCCAATAAATGTCC	21	195	H3 Histone -	*Canis familiaris*	1e-68	TC12182
23	GAAATAAATGCTGCCCT	21	No	-	*-*	-	
24	ACAAATAAAATTGAGCT	21	No	-	*-*	-	
25	CTTACTGCCCCAGCAAT	20	387	Secreted salivary gland peptide	*I. scapularis*	9e-15	TC8005
26	GTGGGCTTCGGGGTCGC	20	157	-	*-*	-	
27	GACTCCAATGAAGGCCC	20	18	Alpha tubulin	*Mus musculus*	0.0	TC9399
28	TTGAGAGGTGGACAGGT	19	53	-	*-*	-	
29	ACGCGACTGTCGCCACC	18	6	Secreted protein	*I. scapularis*	3e-72	TC5798
30	ACGTGACTGTCGCCACC	18	1	-	*-*	-	
31	GGTCCACCCCAGCGACT	17	10	Ribosomal protein L10A	*I. scapularis*	8e-104	TC12372
32	TACTGTACCGAGGCCAG	17	No	-	*-*	-	
33	GTTGTTACGGGTAACGG	17	No	Secreted protein	*I. scapularis*	6e-20	BEAE880TF
34	ATATTGACATTTCGTAG	17	No	Mitochondrial DNA	*R. sanguineus*	0.0	TC12054
35	GCGAGGAGCTTGTCGGG	16	20	-	*-*	-	
36	AAAAAGGCTCAAGAAAT	15	783	-	*-*	-	
37	GGACTCTGTAAGCACCG	15	53	-	*-*	-	
38	CCGGTTCTTTCTTGGTG	15	No	-	*-*	-	
39	GGCGGAATAAAAGCGGT	14	60	60S ribosomal protein L5	*I. scapularis*	4e-146	TC8903
40	ATCTGAGTTTAAACCGA	14	No	-	*-*	-	
41	CCTCCAACGTACTCCGG	14	783	Hypothetical protein	*I. scapularis*	8e-15	TC11473
42	GCACGGCGATGCGACGG	14	16	-	*-*	-	
43	GGTCAGTCGGTCCTTAG	13	783	10kD secreted protein	*A. monolakensis*	5e-28	TC12507
44	TAGGAATTTAAAAGTTG	13	No	Mitochondrial DNA	*R. sanguineus*	0.0	TC5761
45	CTCACCGACCCGTCGGC	13	8	-	*-*	-	
46	CCGAAATAAGGCGAAAC	13	No	-	*-*	-	
47	ACCAGTTCAGGAGAGCC	13	65	-	*-*	-	
48	CCCGGCCACAACCAGGA	13	100	Hypothetical protein	*I. scapularis*	5e-68	TC10088
49	TACGAAGCGCTGGCAGA	12	5	Disulfide isomerase	*Haemaphysalis longicornis*	4e-48	TC8716
50	GGATTTGGTCTCTTTGA	11	783	60S acidic ribosomal protein P1	*I. scapularis*	1e-29	TC13709

^a^ CATG trimmed from each tag’s 5′ end for clarity.

^b^ Indicates tag not found in Control library.

**Table 8:** LongSAGE tags with >10-fold differential expression

**Tag**^**a**^	**Control**	**Infected**	**Difference**	**BmiGI match**			
	**Count**^**b**^	**Count**^**b**^	**(fold)**	**Description**		**Species**	**e-value**
Upregulated with infection							
TGATTGTGTGCTATGTG	1	148	148	No^c^			
GGGGCAAACACTATGGA	0	89	>89	No			
AAGATCACACTGGCATT	1	59	59	No			
TTTTCCCCAACCCAGGA	0	52	>52	BEACP61TR	Microplusin	*R. microplus*	7e-49
TTTTTCCCAACCCAGGA	1	41	41	No			
GGTCAAGGGGTAATAAA	0	38	>38	No			
GTGGTTACGGAGGCGGG	0	38	>38	No			
CAGAAGCTTCAAAGCCA	1	35	35	No			
TCGACAGGGTCATTCCG	0	32	>32	No			
GCCGTTCTTAGTTGGTG	1	30	30	No			
GCGGTTACGGAAGCGGG	0	27	>27	No			
AAGATCACGCTGGCATT	0	25	>25	No			
CACATCATAGAACAGCT	0	23	>23	No			
ACAAATAAAATTGAGCT	0	23	>23	No			
TACTGTACCGAGGCCAG	0	18	>18	No			
GTTGTTACGGGTAACGG	0	18	>18	BEAE880TF	Secreted protein	*I. scapularis*	6e-20
ATATTGACATTTCGTAG	0	18	>18	TC12054	Mitochondrial DNA	*R. sanguineus*	0.0
CCGGTTCTTTCTTGGTG	0	15	>15	No			
AAAAAGGCTCAAGAAAT	1	15	15	No			
ATCTGAGTTTAGACCGA	25	354	14.2	TC5761	Mitochondrial DNA	*R. sanguineus*	0.0
GGTCAGTCGGTCCTTAG	1	14	14	TC12507	10kD secreted protein	*A. monolakensis,*	5e-28
GTAGCCGCCAGCCAAGG	2	23	11.5	No			
CAGGCTGTCCCAGCAAT	4	45	11.2	BEAE009TR	Secreted salivary peptide	*I. scapularis*	1e-05
CTTACTGCCCCAGCAAT	2	21	10.5	TC8005	Secreted salivary peptide	*I. scapularis*	9e-15
Down-regulated with infection							
TCTGGACGCGGCAAGGG	48	1	-48.0	No			
CACGTACAACCTCTGCG	22	0	<-14.0	No			
TTGTGCAGCGATCGGCA	22	1	-22.0	No			
GTACCAGAGGACAAGCC	18	1	-18.0	No			
TCGAACCCCCGGCAGTA	14	0	<-14.0	No			
TGGGGCACGTCCAAGCT	14	1	-14.0	TC6102	Elongation factor beta	*Ornithodoros parkeri,*	1e-85
GTGGTGCCATCGGCGCT	14	1	-14.0	No			
TCGAACCCCCGGCAGTA	12	1	-12.0	TC14104	Ribosomal protein S18	*Ornithodoros parkeri,*	1e-67
GAAGAAGCCATCGGCCG	12	1	-12.0	No			
AACCCCGTCGAGCACCC	12	1	-12.0	TC12306	Ribosomal protein L8	*Glossina morsitans morsitans*	2e-116
GGCCGCTACCCGGACCT	12	1	-12.0	TC10648	Hypothetical protein	*I. scapularis*	1e-93
TGGTGCCCGAAACGAAG	68	6	-11.3	TC12322	Uro-adherence factor A	*Taloromyces stipitatus*	1e-05
AAGAGCGTGTGCGGCTG	36	3	-11.0	TC9377	Hypothetical protein	*I. scapularis*	1e-20

^a^ CATG trimmed from each tag’s 5′ end for clarity.

^b^ To allow comparisons, tag count data normalized to 8,750 total tag counts per library and individual counts adjusted accordingly. Thus, Control Library tags multiplied by 8750/6764 and Infected Library tags by 8750/8750.

^c^ Indicates no match to BmiGI

The only host defense-related protein detected in the SAGE of the infected sample was microplusin, which exhibits antimicrobial activity during oogenesis in *R. microplus*[26]. A tag matching with the microplusin transcript was the fifth-most abundant tag in the *B. bovis*-infected library. However, a microplusin tag was not found in the non-infected library. The rest of the proteins occurring in the infected sample that were annotated had primarily house-keeping functions. Two notable proteins, calreticulin and superoxide dismutase, appeared in the SAGE uninfected library. Calreticulin was up-regulated in ovarian tissue from *B. bovis*-infected ticks in an earlier study [3]. Superoxide dismutase (SOD), a potent antioxidant, is often up-regulated when a cell is experiencing stress and plays a role in the virulence of pathogens [41]. A tag corresponding to SOD was not found in the SAGE infected ovary dataset, and perhaps the absence of this transcript assists in the successful *B. bovis* infection of the tick.

### Overlap between the approaches

**Figure 2:**
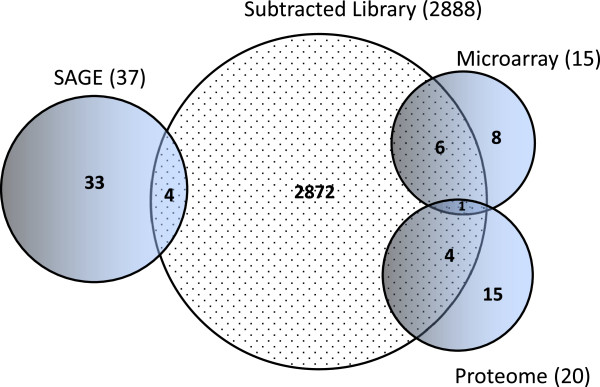
**Overlaps between the differentially expressed transcript datasets.** A total of 2,888, 37, 20, and 15 transcripts make up the datasets from the subtracted library, SAGE, Rachinsky *et al.*[3] proteome and the microarray experiments. The numbers in the intersecting regions of the circles represent the number of transcripts that are common between the represented datasets.

In the three approaches described in this study, the genes found to be up-regulated in response to *B. bovis* infection were compared with each other and with the up-regulated proteins found by Rachinsky *et al.*[3] under the same conditions (Figure 2, Table 9). No overlap was seen between genes detected in the SAGE ovary experiment and those of the microarray and Rachinsky *et al.*[3] proteome study. Only 4 of the 37 transcripts that were differentially expressed in the SAGE protocol were found in the subtracted library dataset. The microarray, subtracted library, and proteome studies compared transcript/protein profiles of infected and uninfected ovary tissues derived from similar conditions (i. e. incubation temperature and time point sampled). In contrast, the SAGE experiment was conducted with the control consisting of engorged females collected and dissected on the day they reached repletion and dropped from the host and the “infected” ovaries from ticks held 6 days post-repletion. Thus, some of the differential expression seen in the SAGE experiment could be strictly related to developmental events in the ovary during this preoviposition stage rather than related to *B. bovis* infection. Another possible confounding factor in the microarray, subtracted library, and the Rachinsky *et al.*[3] experiments was the tick infestations on the uninfected control and the *B. bovis*-infected bovine host took place one month apart due to space limitations in the quarantine facility and an accident that required the euthanization of the control calf. Additionally, the engorgement process of ticks feeding on the infected animal took longer than the engorgement of the ticks feeding on the uninfected control animal. Thus, we adjusted the dissection dates to ensure the females were in similar developmental stages for both groups. This difference in days post-drop before dissection could contribute to developmental differences unrelated to *B. bovis* infection and these might be reflected in the subtracted library and microarray datasets. It also must be noted that infection with *Babesia* alters the serum profile in the affected host as it struggles to cope with the infection [42]. These serum changes are likely causing altered transcription of genes in the tick feeding on *Babesia*-infected host blood. These changes induced by the altered host blood might be confounding the differential transcription study. Differentially expressed genes that we ascribe to *B. bovis* infection of the cattle tick might actually be responding to the altered serum components between the control and infected bovine. Seven transcripts were common to both the microarray and the subtracted library datasets. One of these seven, the Kunitz-type serpin represented by TC9311, was also described in the proteome study of Rachinsky *et al.*[3]. Five other transcripts in the subtracted library also overlapped with overexpressed proteins reported in the ovarian proteome study.

**Table 9:** **Differentially expressed members of BmiGI Version 2.0 in common among subtracted library, SAGE, microarray results and up-regulated proteins from Rachinsky et al. proteome study**[3]

	**Proteome study**	**Microarray**	**SAGE**	**Subtracted library**
Proteome	-	TC9311 Kunitz-type serpin	None	TC8919 Pep.-prolyl *cis-trans* isomerase
				TC8931 Myosin light chain
				TC8950 Calreticulin
				TC9311 Kunitz-type serpin
				TC12119 Cytochrome C oxidase
Array	TC9311: Kunitz-type Serpin	-	None	TC6326 Salivary gland peptide
				TC6491 Kunitz-type protease inhibitor
				TC6758 Protein IscW
				TC9311 Kunitz-type serpin
				TC12551 Salivary gland peptide
				TC13077 Kunitz-type protease inhibitor
				BEABQ71TR GGY domain protein
SAGE	None	None	-	TC6102 Elongation factor beta
				TC9377 Hypothetical protein
				BEACP61TR Microplusin
				BEAE009TR Putative salivary protein
Library	TC8919 Pep.-prolyl *cis-trans* isomerase	TC6326 Salivary gland peptide	TC6102 Elongation factor beta	-
	TC8931 Myosin light chain	TC6491 Kunitz-type protease inhib.	TC9377 Hypothetical protein	
	TC8950 Calreticulin	TC6758 Protein IscW	BEACP61TR Microplusin	
	TC9311 Kunitz-type serpin	TC9311 Kunitz-type serpin	BEAE009TR Putative salivary protein	
	TC12119 Cytochrome C oxidase	TC12551 Salivary gland peptide		
		TC13077 Kunitz-type protease inhib.		
		BEABQ71TR GGY domain protein		

## Conclusion

The complementary experimental approaches in this study produced several differential gene expression datasets associated with the infection of *R. microplus* by *B. bovis*. With the caveats discussed above in mind, transcripts that were detected as differentially expressed by more than one experimental protocol are priority targets for further study of the interactions at the vector-pathogen interface between *R. microplus* and *B. bovis*. A recent report [23] profiled gene expression in *R. microplus* ovarian tissues during feeding, reporting 417 up-regulated ovary-specific transcripts. Their results are not strictly comparable to ours, as our study was designed to detect infection-related differential transcription while [23] was designed to look at overall ovarian transcription during feeding. However, A and G family ABC transporter-, several zinc finger protein-, microcephalin-, cysteine rich secretory protein-, and serine protease-encoding transcripts, among others, were noted in our subtracted library study and that of Stutzer *et al.*[23]. A range of proteases and protease inhibitors were also noted as up-regulated in both studies. It would be interesting to design and conduct a study to discern signaling and regulatory mechanisms that might be occurring on these enzyme systems in the ovary of *R. microplus*. Stutzer *et al.*[23] profiled the adult female transcriptome of *R. microplus*, including that of the ovary, in response to feeding. The study reported here is the first comprehensive profile of the ovarian transcriptome responding to infection. Although a large percentage of tick genes remain without functional annotation, these newly identified gene expression patterns contribute to our understanding of the *R. microplus* transcriptome.

## Competing interests

The authors declare that they have no competing interests.

## Authors’ contributions

AH participated in the bioinformatic analysis of the subtracted library and drafted the manuscript. FDG conceived the study, participated in the design, data collection, and analysis of the study and participated in drafting the manuscript. KGB participated in the data collection, data analysis, and designed and conducted the RT-PCR verification study. LS, SED and CG participated in analysis of the microarray data; GAS participated in the overall study design and collection of tick materials. VN, SED and AD participated in study design and coordinated the sequencing phases. KAB participated in study design and microarray experimental design. All authors read and approved the final manuscript.

## Supplementary Material

Additional file 1 **Unigene dataset from subtracted library sequencing.** This Excel file contains assembled contig and singleton sequences from the subtracted library synthesized from the *Babesia bovis*-infected female tick ovaries (using uninfected tick ovary for the subtraction). Click here for file

Additional file 2 **Relative quantitative real-time PCR primers and probes.** This Word document contains the sequences of the primers and TaqMan probes used in real-time PCR verifications of the microarray results. Click here for file

Additional file 3 **BLASTX annotations of subtracted library unigenes.** This Excel file contains information on the BLASTX analysis of the Unigenes from Additional file 1, including definition line and e-values. Click here for file

Additional file 4 **ORFs for all unigenes as predicted by prot4EST.** Excel file contains the prot4EST predicted open reading frame for each unigene of Additional file 1. Click here for file

Additional file 5 **Unigene GO annotations.** Excel file containing GO terms for Unigenes from Additional file 1 that had corresponding GO classification terms. Click here for file

Additional file 6 **Microarray values for ovary transcripts that were up-regulated in ticks feeding upon a *B. bovis*-infected calf.** FC denotes fold change and d is the SAM statistic. This table in Excel format gives BLASTX definition line and e-value information for members of BmiGI Version 2 that were statistically significantly up- or down-regulated in the microarray experiment. The threshold value delta was set to 0.53 and fold change set to ≥ 2.0 to determine significance. No transcripts were found to be down-regulated under these conditions. Click here for file

Additional file 7 **Overall statistics for SAGE experiment.** Excel file containing raw counts for the SAGE tag libraries, including total number of tags, number of unique tags, and number of mutually occurring tags. Click here for file

Additional file 8 **SAGE tag raw counts in ovary infected tissue and in ovary control uninfected tissue.** Excel file lists each tag and its corresponding number of occurrences for both SAGE libraries. Click here for file

Additional file 9 **SAGE tags with matches to sequences in BmiGI Version 2 in the forward direction.** Excel file lists each SAGE tag that has an exact match to a member of BmiGI Version 2. Click here for file

Additional file 10 **SAGE tags that match sequences in BmiGI Version 2 when tag is reverse complemented.** Excel file lists each SAGE tag that, when reverse complemented, has an exact match to a member of BmiGI Version 2. Click here for file

Additional file 11 **SAGE sequence tags that show higher normalized tag counts in infected samples compared to control samples.** This Excel file contains a list of SAGE tags that show higher normalized tag counts in infected samples compared to control samples. This includes the tag sequence, the normalized tag count for both the control uninfected sample library and the *Babesia bovis*-infected sample library, and the calculated fold-change. Click here for file

Additional file 12 **SAGE tags that show lower counts in infected sample compared to control uninfected sample.** This Excel file contains a list of SAGE tags that show lower normalized tag counts in infected samples compared to control samples. This includes the tag sequence, the normalized tag count for both the control uninfected sample library and the *Babesia bovis*-infected sample library, and the calculated fold-change. Click here for file
