# Supplementary material for: The ovarian transcriptome of the cattle tick, Rhipicephalus (Boophilus) microplus, feeding upon a bovine host infected with Babesia bovis
Source: Parasit Vectors. 2013 Sep 23;6:276. doi: 10.1186/1756-3305-6-276 (PMC4028808; doi:10.1186/1756-3305-6-276)
Supplement: Additional file 2 — Relative quantitative real-time PCR primers and probes. This Word document contains the sequences of the primers and TaqMan probes used in real-time PCR verifications of the microarray results. [file 1756-3305-6-276-S2.docx]

**Table 1: Relative Quantitative** **Real-Time PCR Primers and Probes**

EST^a^ Primers^b^ TaqMan Probe

TC9020 FW 5’ GAACGAACATCTCAGCCACAATG 3’ 5’ FAM-CTGCCGACTCCAAGACCACCACC-TAMRA 3’

RV 5’ ACCTCCGAGACCTGCACTTC 3’

TC9311 FW 5’ CTACGTGCCTCGCTACTTCTAC 3’ 5’FAM-ACACGACCACCAAGTTCTGCGAGCG-TAMRA 3’

RV 5’ TATCCGACTTTGCAGGTCTTCAG 3’

TC13077 FW 5’ ACCCTGCTACGGCTACTTCC 3’ 5’ FAM-AACACGACGACCAACACCTGCGAGC-TAMRA 3’

RV 5’GCACTTGGTCTGGCACTGG 3’

18S FW 5’ CCTGAGAAACGGCTACCACATC 5’ FAM-AGGAAGGCAGCAGGCGCGC-TAMRA 3’

RV 5’ GTGCCGGGAGTGGGTAATT 3’

^a^ Based on BmiGI Version 2 designations (http://compbio.dfci.harvard.edu/tgi/cgi-bin/tgi/gimain.pl?gudb=b_microplus)

^b^ Forward (FW) & reverse (RV)
